# Exploratory Study on Screening Dementia Based on Frontal Polar Cognitive Activation Using Portable Wireless Functional Near-Infrared Spectroscopy

**DOI:** 10.7759/cureus.85902

**Published:** 2025-06-13

**Authors:** Atsumichi Tachibana, Shun Irie, Yumie Ono, J Adam Noah, Muneto Tatsumoto, Daisuke Taguchi, Nobuko Tokuda, Shuichi Ueda

**Affiliations:** 1 Department of Anatomy, Dokkyo Medical University, Mibu, JPN; 2 Division for Smart Healthcare Research, Dokkyo Medical University, Mibu, JPN; 3 Department of Electronics and Bioinformatics, Meiji University, Kawasaki, JPN; 4 Department of Psychiatry, Yale School of Medicine, New Haven, USA; 5 Department of Neurology, Dokkyo medical university, Mibu, JPN; 6 Department of Judo Therapy, Teikyo University, Utsunomiya, JPN; 7 Long-Term Care Health Facility for the Elderly, Cosmopia, Mito, JPN

**Keywords:** dementia screening, fnirs, frontal-polar cortex, mild cognitive impairment, near-infrared spectroscopy, neuroscientific assessment, portable wireless fnirs, prefrontal cortex

## Abstract

Objectives

The aims of this study were to examine whether a portable wireless functional near-infrared spectroscopy device ([fNIRS] Hb-13-2, Astem Co., Ltd., Kanagawa, Japan) could potentially be used as a simple and minimally burdensome tool for screening cognitive function in older adults, particularly for identifying dementia-related changes, and to explore the feasibility of establishing a methodological framework for such assessment.

Methods

Subjects with dementia (n=10) and cognitively intact subjects (n=10) over 60 years of age were asked to perform a simple arithmetic task (Uchida-Kraepelin psychodiagnostic [UKP] test: 30 s × 5 trials) while undergoing frontal polar hemodynamic monitoring.

Results

Significantly increased changes in oxygenated hemoglobin (oxyHb) in Fp1 and Fp2 on the frontal polar cortex (FPC) during the task were more pronounced in the dementia subjects than in the cognitively intact subjects. In addition, the peak increase in oxyHb in cognitively intact subjects occurred in the first half of the task period, whereas it occurred in the second half of the task period in subjects with dementia. In all subjects, the relationship between increase/decrease of oxyHb in Fp1 and Fp2 and the UKP test results showed a positive correlation (R=0.307 and R=0.386, respectively).

Conclusion

The pattern and timing of hemodynamic responses in the FPC measured by portable fNIRS during the UKP test may have potential as a non-invasive screening indicator for cognitive decline.

## Introduction

Dementia is a major public health issue, particularly in developed countries, where it affects a large proportion of the aging population and poses significant social and medical challenges [1]. Alzheimer’s disease is the most common type, accounting for approximately 60% of all cases, followed by cerebrovascular dementia at 20-30%. Dementia is characterized by progressive memory impairment and cognitive decline, often accompanied by disorientation, impaired recognition, and functional disability due to neurodegeneration in regions such as the hippocampus [2]. Patients may also experience disorientation in time and place, impaired recognition of people, and physical disability.

Standard clinical assessments for dementia, including the Mini-Mental State Examination (MMSE) and the Montreal Cognitive Assessment (MoCA), are widely used tools that rely on the subjective evaluation of psychological and behavioral responses [3,4]. However, these tests only provide indirect insights into brain function and are limited in their ability to capture underlying neural activity [5,6]. To address these shortcomings, neuroimaging techniques such as functional magnetic resonance imaging (fMRI), electroencephalography (EEG), and conventional functional near-infrared spectroscopy (fNIRS) have provided valuable insights into brain function [2,7-9]. However, these modalities are generally expensive, require bulky equipment, and involve complex procedures, limiting their practical use in routine clinical settings. In contrast, the portable wireless fNIRS system employed in this study is compact, cost-effective, and easy to handle, offering a promising alternative for clinical applications.

In this study, we aimed to establish a feasible, objective, and simplified screening method for dementia using a portable wireless fNIRS system in combination with the Uchida-Kraepelin psychodiagnostic (UKP) test as a simple arithmetic task. The UKP test involves repeated single-digit additions that rely on attention and short-term memory, cognitive domains frequently impaired in dementia. These types of simple arithmetic tasks are not only easy to administer but also elicit activation in the frontal pole and inferior frontal gyrus, areas known to be associated with attentional control and mathematical reasoning [10]. Moreover, prior studies have suggested that mathematical priming is associated with broad social and cognitive functions that affect quality of life (QOL) [11,12]. Therefore, we hypothesized that combining portable fNIRS with a basic cognitive task such as the UKP test could provide a practical method for identifying prefrontal cortical dysfunction in subjects with dementia. This study evaluates the potential of such a combined approach for use as a new, non-invasive dementia screening tool suitable for routine clinical use.

A portion of this article was previously presented as an abstract and poster at the APPW2025 conference on March 18, 2025.

## Materials and methods

Participants

This study included data from 10 subjects with dementia (age 85.3 ± 7.9 years), including mild cognitive impairment, and 10 cognitively intact subjects (age 67.5 ± 4.6 years). These subjects were able to add single digits and write down the answer. Subjects with dementia were recruited from patients who visited the Department of Neurology, Dokkyo Medical University (Mibu, Japan), and from residents and commuters of the geriatric health care facility “COSUMOPIA” (Mito, Japan). Cognitive function in the dementia group was assessed using the MMSE, which was administered by trained clinical staff. Subjects with mild-to-moderate cognitive impairment, as indicated by MMSE scores, were included. There were no exclusion criteria, but the participating patients had no symptoms of visual dysfunction associated with hemianopsia or hemispatial neglect. Written informed consent was obtained from the participants after the purpose of the experiment was explained. All experiments were conducted in accordance with the tenets of the Declaration of Helsinki and were approved by the Ethics Committee for Experimental Research on Human Beings and Dokkyo Medical University Hospital (Approval No. R-55-6J). This study was conducted during the application periods of the Japan Society for the Promotion of Science KAKENHI Grant No. 20K11262 (April 2020 to March 2025) and the Dokkyo International Medical Education and Research Foundation Research Grant Award 2024 (April 1, 2024 to March 31, 2025). The study aimed to recruit as many participants as possible, based on the premise that the required statistical significance could be achieved within this timeframe. As a result, 12 subjects in the cognitively intact group and 12 subjects in the dementia group participated in the study, but fNIRS data from two subjects in each group could not be recruited because there were noisy fNIRS data that could not be used due to excessive body movements or contamination with ambient light and also because some subjects could not perform the UKP test continuously. The final sample sizes in the cognitively intact group (n = 10) and the dementia group (n = 10) were considered adequate for group-level comparisons, as they are comparable to or greater than those used in previous fNIRS studies investigating cortical activation during cognitive-behavioral tasks [13,14]. For correlational analyses with UKP test scores, data from both groups were pooled (n = 20) to increase statistical power. This combined sample size is also consistent with those used in prior studies performing similar correlation analyses [15,16].

fNIRS device and the signal measurement

**Figure 1:**
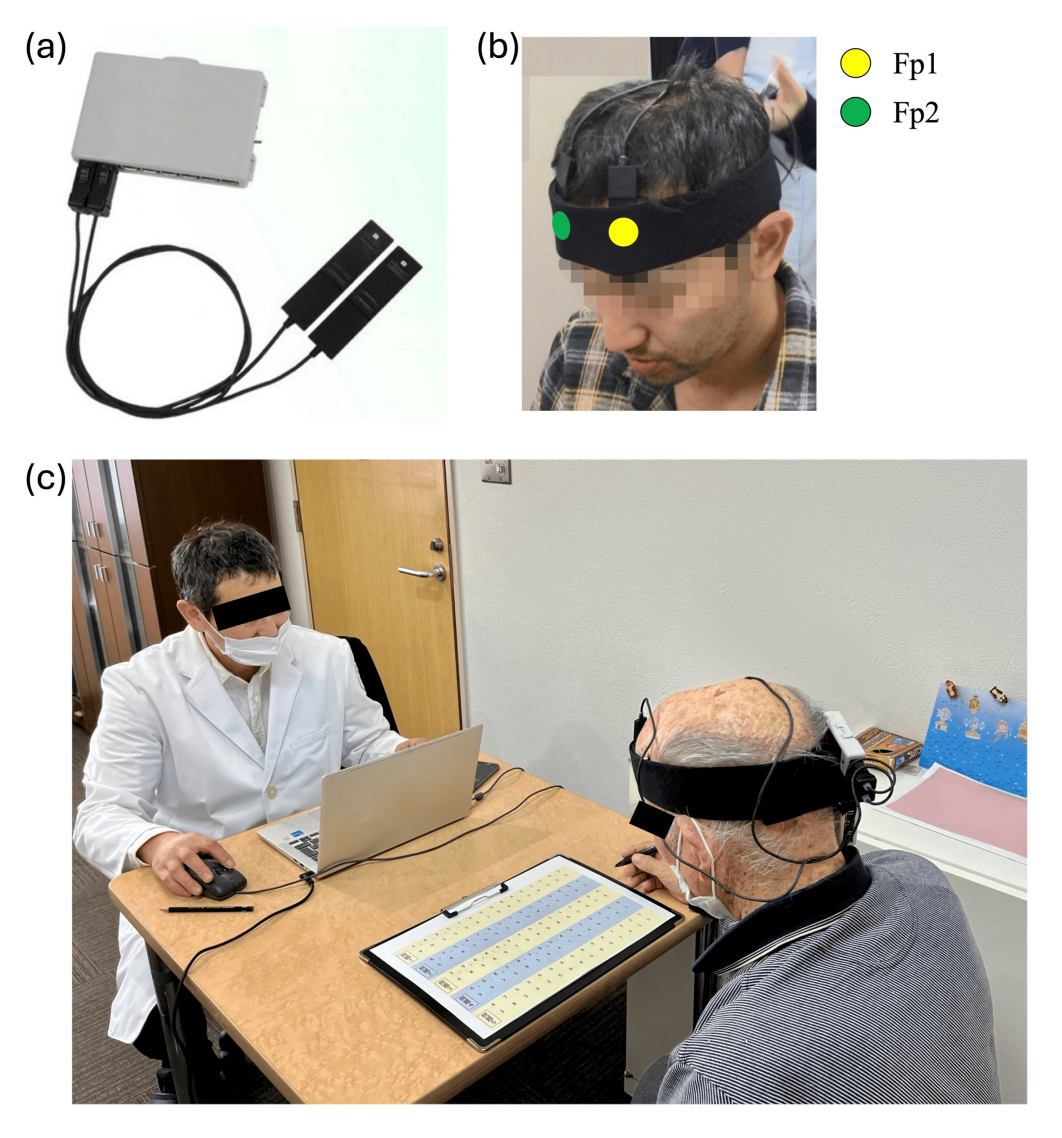
Portable wireless fNIRS device and the experimental setting (a) Portable wireless fNIRS device used in this study (Hb-13-2; Astem, Kawasaki, Japan). (b) Experimental layout with Hb-13-2 attached on Fp1 and Fp2. The subject shown in the photograph has provided written informed consent for the publication of his image. (c) Experimental condition. The researcher in the white coat is the corresponding author, and the individual shown from an oblique back view while wearing the fNIRS device is a study participant. Both individuals provided written informed consent for the publication of their photographs. To protect identity, all photographs have been anonymized by masking the eyes. As these images are used solely for illustrative purposes and do not constitute research data, approval by the institutional review board was deemed unnecessary according to our institutional guidelines. fNIRS, functional near-infrared spectroscopy device

The near-infrared spectroscopy (NIRS) signal was measured using an Hb-13 optical brain function measuring device (Astem Co., Ltd., Kanagawa, Japan). Figure 1 shows an overview of the apparatus and settings used to measure NIRS signals. Hb-13 is a portable wireless fNIRS device using Bluetooth in a hair-band style. The probe is small and lightweight (approximately 10 g) so as not to be a burden on the subject and can be quickly and easily attached to the forehead area corresponding to Fp1 and Fp2 of the subject. The device consists of two embedded light-emitting diodes (LEDs) with two wavelengths of 830 ± 10 nm and 770 ± 10 nm as light sources and two silicon PIN photodiodes as light detectors. The light sources and detectors are spaced 35 mm apart, with a channel between the LEDs/photodiodes. The average light output is 1.0 mW at both 780 nm and 840 nm at the surface of the light source, allowing for safe, non-invasive brain function measurement through cerebral blood flow. Though this device has already been reported in the assessment of prefrontal cognitive function [17], the frontal polar cortex (FPC) of the prefrontal cortex (PFC) was chosen as the region of interest because the cognitive task used in this study is thought to activate the frontal lobe [11]. The head module was mounted so that channels 1 and 2 were positioned directly over Fp1 and Fp2 in the FPC, respectively, according to the international 10-20 system of EEG. Oxygenated hemoglobin (oxyHb) and deoxygenated hemoglobin (de-oxyHb) were measured in the two channels with a sampling interval of 0.2 s.

Experimental procedure for the evaluation of UKP test with fNIRS

For the UKP test experiment with the portable wireless fNIRS, a block design comprising five trials was employed. The experiment included 30-s rest periods staring at a crossed solid fixation point and 30-s task periods for the UKP test. The subjects wore the fNIRS probe on their foreheads at the Fp1 and Fp2 positions. The UKP test comprises answering only the number of first places by adding two adjacent numbers from the left on a form with a random list of single-digit integers 3, 4, 5, 6, 7, 8, and 9. The testee is required to calculate as quickly and accurately as possible, and the number of answers (correct answers) is recorded. The UKP test is originally a task test method to measure working behavior in terms of ability and personality/behavioral characteristics [18], but in this study, it was employed as a cognitive task to elicit activation of the FPC, which is involved in sustained concentration and attention, over a period of 30 s [19]. Each subject practiced sufficiently and then performed the real UKP test with NIRS measurements.

All experiments were conducted in the same room under identical lighting conditions for all participants. To minimize noise, a designated experimental room isolated from external disturbances was used. Prior to the experiment, all participants received both written and verbal instructions as outlined in the information sheet submitted to the institutional ethics committee. These standardized instructions ensured consistent understanding of the task content and the experimental flow across participants.

Considering the ease of handling in clinical interviews and utility in elderly people and people with dementia, both the crossed solid fixation point during the rest period and the UKP test during the task period were administered on paper. Although there is a loss of about 1 s or less when switching the paper used for the rest and task periods, the fNIRS-measured blood flow dynamics inherently lag neural activity by a few seconds and fluctuate slowly; thus, this manual switching is unlikely to affect the fNIRS signal.

fNIRS data processing and analysis

The data measured by fNIRS (moving average; 3) were used as brain functional blood flow data (oxyHb and de-oxyHb) using the skin blood flow separation method with multiple probe placement method and the brain functional and systemic blood flow variability separation method with the hemodynamic separation method developed by Yamada et al. [20]. For each subject, the average oxyHb and de-oxyHb data were analyzed during five 30-s blocks of the UKP test task. A 10-s rest period was allowed before task onset, and a 20-s rest period was allowed after task offset. The oxyHb and de-oxyHb values obtained from NIRS-SPM were filtered (low-pass filtered using the hemodynamic response function [HRF] with correction for serial correlations) to be used as a time-series hemodynamic signal [21]. In addition, baseline drifts were corrected using wavelet minimum description length (Wavelet-MDL) detrending functions provided by the NIRS-SPM software package [22]. A generalized linear model (GLM) analysis based on the HRF was performed using NIRS-SPM to compare between groups of participants. The GLM analysis enables decomposition of the signal into brain-derived hemodynamic responses and residuals. The coefficients for the hemodynamic response, referred to as β values, are interpreted as indicators of brain activity. The parameters for Wavelet-MDL and HRF were set to the default values embedded in the NIRS-SPM software [21,22], ensuring methodological consistency. Correlation coefficients of the two parameters by β and the UKP test results were calculated. A correlation coefficient of R = 0.3 or greater was defined as positive correlation.

## Results

Score for the UKP test

To investigate any differences in the ability to process computational tasks that could influence neural responses in the FPC of the PFC, the number of responses (number of correct answers) to the UKP test was measured in 10 cognitively intact subjects and 10 subjects with dementia (including those with mild cognitive impairment). The mean MMSE score of the dementia group was 24.2 ± 4.18, indicating mild cognitive impairment. The mean number of correct responses by the UKP test score in the cognitively intact and dementia groups was 120.40 ± 49.58 and 42.70 ± 22.38, respectively. A paired t-test revealed a significant difference in the number of responses to the UKP test between the two groups (p = 0.001).

Event-triggered averages for task versus baseline

**Figure 2:**
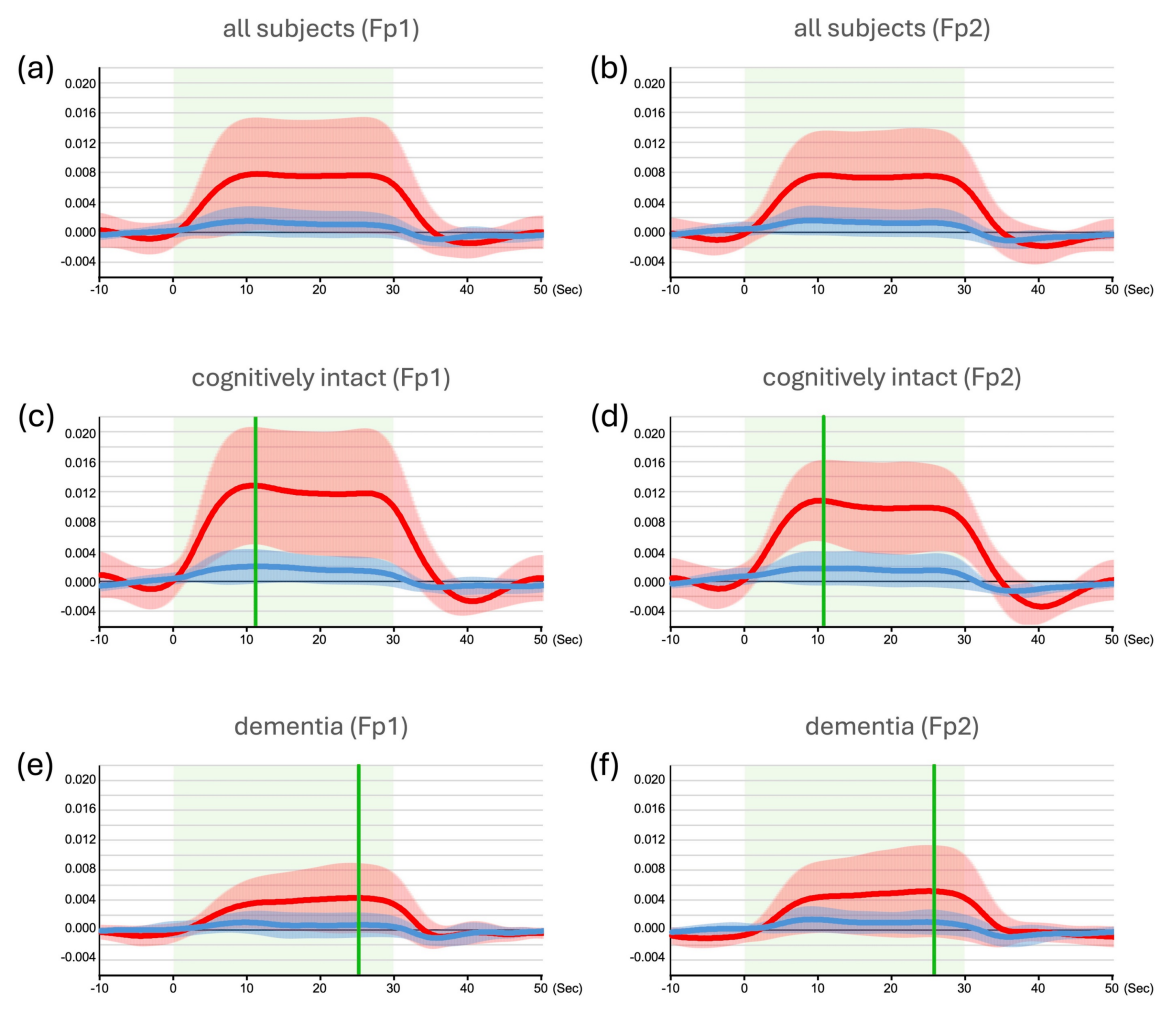
Averaged hemodynamic responses in the FPC during the UKP test (a, b) Averaged hemodynamic responses in the Fp1 and Fp2 regions, respectively, during the UKP test in all subjects (n = 20). Time courses of oxyHb and de-oxyHb are shown as red and blue lines, respectively. Shaded areas in light red and blue represent ±SD for oxyHb and de-oxyHb, respectively. Light green areas indicate the task duration. (c, d) Averaged hemodynamic responses in the Fp1 and Fp2 regions, respectively, for cognitively intact subjects (n = 10). Red and blue lines represent oxyHb and de-oxyHb responses, with shaded areas indicating ±SD. Light green shading denotes the task period. Green vertical lines indicate the time points at which the peak oxyHb values occurred, notably in the first half of the task durations. (e, f) Same format and conditions as in panels (c) and (d), but for subjects with dementia (n = 10). In this group, peak oxyHb values (green vertical lines) appear later within the task duration. FPC, frontal polar cortex; de-oxyHb, deoxygenated hemoglobin; oxyHb, oxygenated hemoglobin; SD, standard deviation; UKP, Uchida–Kraepelin psychodiagnostic test

Figures 2a, 2b show plots of the averaged activation patterns for oxyHb and de-oxyHb on Fp1 and Fp2 during the UKP test in all 20 subjects. Significant activity versus baseline was observed for tasks in oxyHb responses in both hemispheres (p < 0.001). Figures 2c, 2d show the plots of the averaged activation patterns for oxyHb and de-oxyHb in Fp1 and Fp2 during the UKP test in the cognitively intact, and Figures 2e, 2f show those in dementia groups, respectively. Significant activity versus baseline averages was observed in oxyHb responses (Figures 2a-2f, p < 0.001). The significant increase in oxyHb in cognitively intact subjects was greater than that in subjects with dementia (p < 0.001). In terms of oxyHb dynamics, cognitively intact subjects showed a peak increase early in the task period, while subjects with dementia showed a peak at the end of the task period (Figures 2c-2f).

Correlation between a simple calculation task and the hemodynamic signals

**Figure 3:**
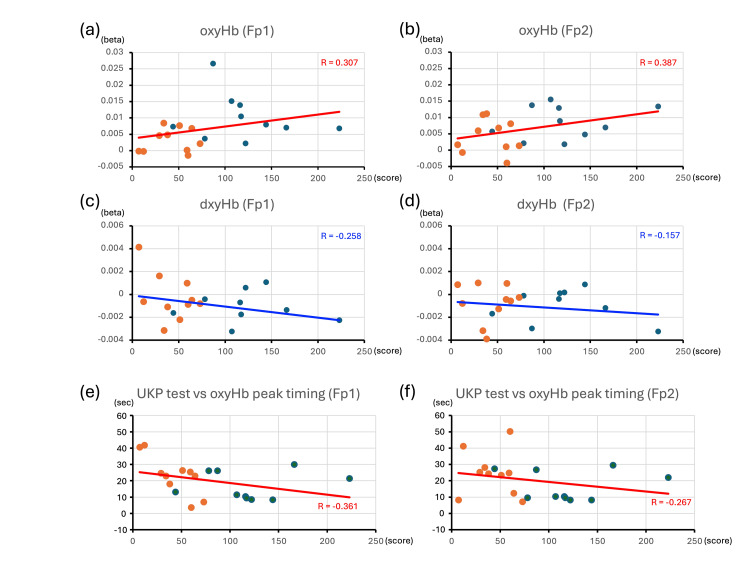
Correlation between the UKP test score and the hemodynamic responses (a–d) Correlation coefficients between the averaged amplitudes of oxyHb or de-oxyHb in the Fp1 or Fp2 regions and UKP test scores. Orange dots and corresponding linear regression lines represent subjects with dementia, while dark blue dots represent cognitively intact subjects. Note the positive correlation between the averaged oxyHb amplitude and UKP test scores (R > 0.3). (e, f) Correlations between the timing of the peak oxyHb increase during the task period and UKP test scores, shown separately for each subject group. Orange dots and regression lines indicate subjects with dementia, while dark blue dots indicate cognitively intact subjects. Red lines in each graph represent regression lines for all subjects combined. Note the negative correlation in the Fp1 region (R = –0.361). de-oxyHb, deoxygenated hemoglobin; oxyHb, oxygenated hemoglobin; UKP, Uchida–Kraepelin psychodiagnostic test

Finally, we assessed the correlation between the hemodynamic signals (β values for oxyHb and de-oxyHb) during the task and the scores from the UKP test in all 20 subjects (Figures 3a-3d). Positive correlations were found for oxyHb in Fp1 and Fp2 (R > 0.3) (Figures 3a, 3b). No noticeable correlation coefficients were shown in those for de-oxyHb, but an inverse correlation with Fp1 and Fp2 in the dementia group was found (Figures 3c, 3d). As shown in Figures 2c-2f, the peak increase in oxyHb in Fp1 and Fp2 for the cognitively intact group occurred in the first half of the task performance, while that of the dementia group occurred in the second half. Therefore, we explored the correlation between the timing of the peak increase in oxyHb during the task period and the UKP test scores (Figures 3e, 3f). The findings revealed a negative correlation in Fp1 (R = -0.361) (Figure 3e), i.e., for all subjects in both groups, there was a correlation between an earlier peak increase in oxyHb and a higher UKP test score in Fp1. However, a weak negative correlation was found in Fp2 (R = -0.267).

## Discussion

The purpose of this study was to verify whether portable wireless fNIRS could be used to screen for dementia in a simple and objective manner without burdening the subjects and to evaluate and confirm the usefulness of the device. To provide a simple measure of cognitive function by the portable wireless fNIRS, we used the UKP test, which is a simple arithmetic task. Exact calculation requires explicit rote memorization of previously learned answers, such as computing the answer to a small addition or multiplication problem (e.g., 3 + 4 = 5 or 7). Because the answers are concrete answers stored in memory, learning is item-specific, and extensive training on a subset of addition problems reduces reaction time to these specific problems, but this reaction time advantage does not extend to new, untrained problems; for example, it includes complex mathematical tasks that most people do not use in everyday life [12,23]. Thus, the UKP test with the task of adding single-digit numbers to each other is considered very suitable for objective dementia determination in that the task is something that everyone utilizes daily and the outcome of the task is left to reaction time to recall memories. Exact calculation has been reported to activate the frontal pole (including the inferior frontal gyrus) network, which is involved in attentional control processes such as inhibition and selection and is particularly important when processing linguistic problems [10]. Furthermore, mathematical priming power has been shown to affect both social and cognitive processing related to QOL, especially social functioning [11,12]; therefore, it may be suitable for screening for dementia in this regard as well. For these reasons, we chose math tasks, which should be strongly related to the cognitive status of dementia, as the task for this study. The results showed that the cognitively intact subjects showed large increases in oxyHb in Fp1 and Fp2 while performing the UKP test, but subjects with dementia did not show the same increase in oxyHb. This result suggests that the UKP test requires sustained concentration during the task period, facilitating activation of the FPC, and that the subjects with dementia did not show much increase in blood flow in Fp1 and Fp2 with decreased performance compared to cognitively intact subjects. In addition, the peak increase in oxyHb in the cognitively intact subjects occurred early in the task, whereas it occurred toward the end of the task in those with dementia. Ono et al. [14] reported that blood flow patterns in the FPC differed between experts and novices in the cognitive task, and it has also been reported in recent years that differences in cerebral blood flow in each cortical area and the timing of its change differ according to the skill and cognitive state of the subject, even when performing the same task [13,15,16]. The present study found similar results: correlations for lower MMSE scores reached a later peak oxyHb at Fp1 during the task period in the dementia group, while there was no correlation in the cognitively intact group. These results suggest that the FPC processes the task differently based on cognitive status. Although these interpretations remain exploratory, we hypothesize that the activation of the FPC peaks shortly after the start of the task in cognitively intact individuals as they develop a strategy, which then gradually decreases as they adapt to the task; however, individuals with dementia may struggle to establish a strategy early on, resulting in later peak activation. Regarding the timing and dynamics of oxyHb peaks observed in this study, they are considered within the context of neural adaptation to task demands in aging populations. Previous fNIRS studies have reported altered prefrontal hemodynamic responses during cognitive tasks in older adults [24-26], supporting the plausibility of our interpretation. Thus, the advantage of the higher temporal resolution of fNIRS over fMRI is that detailed changes in blood flow patterns can reveal behavioral differences between subjects (the sampling interval for Hb-13 used in this study was 0.2 s) [27]. Furthermore, the correlation between the mean hemodynamic response during the task and task performance for all subjects in both groups showed a positive correlation with oxyHb in Fp1 or Fp2 and the number of correct answers on the UKP test (R=0.307 and 0.386, respectively). These exploratory findings suggest the potential utility of examining the rate and timing of oxyHb changes in response to task performance, along with behavioral indices, for identifying differences in cognitive function. We cautiously propose that such patterns may serve as a basis for future studies on neuroscientific screening approaches for dementia.

On the other hand, the MMSE is one of the most used dementia screening methods since its creation in 1975 [3]. The MMSE is a simple test designed for subjects suspected of having Alzheimer’s disease or other forms of dementia and is administered to subjects in the form of verbal questions scored on a 30-point scale, with a score of 30 to 27 indicating no abnormality, 27 to 23 indicating suspicion of mild cognitive impairment, and 23 or less indicating suspicion of dementia. While this is a classic screening method, it is not objective, as it can vary greatly depending on the mood and condition of the subject on any given day or on the person asking the questions. The method used in the present study, which combines behavioral assessments with portable wireless fNIRS measurements, may offer a valuable approach to examining brain function in individuals with suspected cognitive decline [11]. The observed association between changes in cortical activity and UKP test scores suggests the potential for identifying factors related to cognitive function. Fujimaki et al. reported on the use of fNIRS in assessing QOL using a short-form questionnaire, affective symptoms, apathy, feelings of stress, and task performance using the number of responses in a serial arithmetic task [11]. The study suggested that abnormal cortical activation as measured by fNIRS is associated with social functioning in a neuropsychological profile. In addition, Koechlin and Hyafil reported that the FPC is effective in protecting the execution of long-term mental plans from immediate environmental demands [28]. We have also suggested that an important aspect of cognitive decline in the FPC activation relevant to social functioning, such as in dementia, is also attributable to executive function [29].

The portable wireless fNIRS used in this study is the Astem NIRS (Hb-13 series), a relatively inexpensive functional brain imaging device, starting at JPY ¥180,000, that can measure the hemodynamic response in the PFC. The Bluetooth-LE wireless system and hair-band probe allow measurement of the prefrontal hemodynamic response in a more natural environment, freeing subjects from the inconvenience of wires and the discomfort of wearing a large device on their head. Testing cognitive function under natural conditions is particularly important for subjects with dementia, as it helps reduce the burden of assessment and provides objective neuroscientific measures. This approach is also beneficial for cognitively intact subjects and diagnosticians [27]. It is also a very simple method for both the patient and diagnostician, as the task used is paper-based (the rest period consists only of placing a crossed fixation point printed on paper) and does not require any special computer application, touch-panel screen, or mouse. We cautiously suggest that portable wireless fNIRS holds promise as a simple, objective, and low-burden method for supporting exploratory research into dementia screening in clinical practice.

Limitations

In this study, the mean age of cognitively intact subjects (67.5 years) was 17.8 years younger than that of the subjects with dementia (85.3 years). This age gap reflects the demographics of recruitment settings, as cognitively intact participants were primarily active employees at Dokkyo Medical University Hospital and the geriatric care facility “COSUMOPIA.” While significant group differences in hemodynamic responses and correlations with UKP test performance were observed, future studies should aim to age-match groups more closely to improve the reliability of comparisons. Although potential learning effects could influence the results, participants were given sufficient practice time before performing the UKP test to minimize this effect. Moreover, all participants were Japanese, and Japan has a near 100% literacy rate, making it unlikely that literacy levels significantly affected the findings. Comorbidities, which may affect neurophysiological measures, were not fully controlled and should be considered in future research.

Another limitation of this study is the relatively small sample size (n = 10 per group). To address the potential for overinterpretation based on small subgroups, regression lines were calculated and presented only using the total sample (n = 20). This approach reduces the influence of outliers and enhances the stability of observed trends. Furthermore, previous studies using fNIRS to assess FPC activation have reported similar findings with comparable sample sizes (e.g., n = 20), supporting the methodological validity of this study [14-16].

Nevertheless, we acknowledge the risk of type I errors, particularly due to multiple correlation analyses conducted. To address this, we avoided making strong causal interpretations and instead emphasized the exploratory nature of our findings. Furthermore, given the small sample size, the possibility of type II errors (i.e., failing to detect subtle but meaningful effects) cannot be ruled out. Future studies with larger sample sizes will be essential to increase statistical power and verify these preliminary results.

Furthermore, several subjects with dementia who had low MMSE scores had difficulty fully understanding the test procedure when completing the UKP test and therefore required assistance from a carer. One cognitively intact subject had a UKP test score at a level comparable to that of a subject with dementia, but we did not assess this subject with a dementia screening test such as the MMSE or MoCA-J. As these subjects did not perform the UKP test independently, the possibility of errors in the test results and hemodynamic signal data cannot be excluded. However, compared to the trends in the results of this study and previous studies of prefrontal activation patterns during cognitive tasks, it is predicted that these factors are not likely to produce such large errors and that more reliable studies can be conducted in the future by expanding the amount of data to be obtained.

## Conclusions

The objective of this study was to evaluate whether a portable wireless fNIRS device, combined with the UKP test, could detect differences in prefrontal hemodynamic responses between individuals with and without dementia. Subjects with dementia exhibited greater increases in oxyHb levels in the Fp1 and Fp2 regions during the task compared to cognitively intact participants. Moreover, the peak oxyHb increase tended to occur earlier in cognitively intact individuals and later in those with dementia. These findings suggest that measurements obtained using a portable wireless fNIRS device during the UKP test may serve as a simple and objective approach for screening cognitive function, particularly in identifying dementia-related patterns of neural activation. Further studies involving larger and more diverse populations in clinical settings are necessary to validate this approach.
